# Formation mechanism of the quarantine hotel booking intention of potential consumers

**DOI:** 10.3389/fpsyg.2022.997667

**Published:** 2022-09-28

**Authors:** Guihua Wu, Yanwen Wang, Xuejia Li, Meizhen Lin

**Affiliations:** College of Tourism, Huaqiao University, Quanzhou, Fujian, China

**Keywords:** quarantine hotel, booking intention, potential consumer, corporate social responsibility, COVID-19

## Abstract

In this paper, we investigated the quarantine hotel (QH) booking intention (BI) of potential consumers from a corporate social responsibility (CSR) perspective. Mixed methods were adopted to explore the formation mechanisms of QH BI of potential consumers when the COVID-19 pandemic recedes. In Study 1, we constructed a theoretical model of QH BI of potential consumers based on grounded theory and put forward research propositions. In Study 2, we tested the robustness of the model and identified the mediating effect through two situational experiments. The research results showed that: (1) Potential customers are more willing to book QHs than normal hotels (NHs). (2) Multiple mediating mechanisms are involved in the effect of the operation as a quarantine facility on BI of potential consumers, including “QH-corporate social responsibility (CSR)-customer trust (CT)-BI” and “QH-CSR-customer gratitude (CG)-BI.” This research not only has theoretical significance for deepening and expanding social exchange theory and hotel CSR theories but also provides guidance for the participation of the hotel industry in the prevention and control of the COVID-19 pandemic and hotel marketing after the pandemic ends.

## Introduction

As COVID-19 continues ravaging the world, the global hospitality industry has reached a near standstill and the rapidly declining occupancy rates have become a major concern for hotel industry executives (Foroudi et al., 2021) Hospitals face problems such as overcrowding, inadequate quarantine space, and bed shortages with the continuous increase in COVID-19 positive cases (Teng et al., 2020). Since hotels and hospitals share many common features, such as separate rooms and toilets, catering and cleaning services, and skilled service staff (Teng et al., 2021), scholars have proposed to adapt hotels into temporary quarantine facilities to meet the surging demand for temporary quarantine accommodations (Ramírez-Cervantes et al., 2020). Thus, quarantine hotels emerged as designated quarantine facilities temporarily requisitioned by the government.

In COVID-19 prevention and control practices, different hotels have engaged in the role of quarantine hotels to different extents. Some hotels provided quarantine services in an attempt to obtain government subsidies to compensate for economic losses during the pandemic and show their willingness to undertake social responsibility to win the recognition of potential consumers (Handani et al., 2022). However, some hotel managers chose to abandon the quarantine business, which, in their opinion, would lead to additional labor costs arising from a cumbersome renovation process. In addition, they think that if they take the quarantine business, potential consumers would label their hotels as dangerous sites (Dincer and Gocer, 2021) and change booking decisions, leading to a loss in market share. Whether potential consumers will change their reservation intention because of the hotel’s undertaking of corporate social responsibilities (CSRs), such as serving as quarantine hotels?is a research topic with both theoretical and practical value and needs to be revisited.

Although the factors affecting hotel consumers’ reservation intention have been extensively investigated, few studies focused on how undertaking CSRs such as serving as quarantine hotels in a major crisis affects potential consumers’ reservation intention. In the existing literature, scholars have studied the positive effects of different CSRs, including charitable donations, engagement in environmental protection, and sustainability commitments, on consumers’ behavioral intentions (Ahn et al., 2020; Liu et al., 2020; Kapoor et al., 2022). Unlike CSRs covered in previous studies, undertaking COVID-19 quarantine hotel operations may bring adverse effects such as raising hotel operating costs, endangering the physical and mental health of staff, and impacting hotel brand reputation. In this case, will potential consumers still book hotels that have served as quarantine hotels? In addition, existing studies on quarantine hotels found inconsistent results on the effect of hotels’ undertaking of social responsibility on potential consumers’ reservation intention during COVID-19 (Shin et al., 2021; Tong et al., 2021), suggesting that the internal mechanism remains to be explored. Considering the tremendous impact of COVID-19 on the hotel industry, this study finds it necessary to explore the mechanisms by which quarantine hotels’ undertaking of CSRs affects potential consumers’ reservation intention.

To fill the research gap, the present research focuses on whether potential consumers regard the operation as a quarantine facility as a CSR behavior of QH and how CSR affects their BI after COVID-19 is over, aiming to fully show the effect of hotel property (QHs vs. normal hotels (NHs)) on BI of potential consumers and the involved mediating mechanisms. Contributions of this research are as follows: We explained the formation mechanism of the QH Bi of potential consumers from the perspective of social exchange theory; theoretically, this paper enriches the theoretical study on CSR in the hospitality industry. In addition, this research suggests that engaging in pandemic prevention and control activities, which is a way to contribute to society, manifests the willingness of hotels to undertake social responsibility and can make them the favored choice of consumers; in practice, this paper provides a marketing strategy for managers of QHs and can guide the development of QHs in the post-pandemic era.

## Literature review

### Quarantine hotel

Quarantine hotels, also known as quarantine centers for medical observation, are designated quarantine sites temporarily requisitioned by the government to prevent the continued spread of COVID-19 in the present and future time. These quarantine hotels provide accommodation and quarantine spaces for medical staff, suspected cases, travelers, and returning guests. They also provide accommodation rooms, catering services, personal protective equipment, and simple clinical monitoring (Jordan-Martin et al., 2020; Teng et al., 2020).

Since the outbreak of COVID-19 in 2019, researchers have studied quarantine hotels from public health and business management perspectives. From the public health perspective, the existing studies have focused mainly on the effectiveness of quarantine hotels against the pandemic, the construction of quarantine spaces, and public health management experiences (Goei et al., 2020; Dincer and Gocer, 2021; Fotheringham et al., 2021). For example, the COVID-19 prevention and control experience in Australia and Singapore have shown that care centers consisting of quarantine hotels and other facilities are effective in preventing the wide spread of COVID-19 in the region (Goei et al., 2020; Fotheringham et al., 2021). From the business management perspective, academic research on QHs mainly focuses on two groups: hotel staff and residents. Some studies have suggested that QH staff face a higher risk of infection when hosting guests who need to be quarantined, and therefore their psychological state at work (fear of COVID-19, work stress, financial anxiety, etc.) and motivation to continue working deserve attention (Goh and Baum, 2021)Some studies have investigated the hotel experience, psychological state, and evaluation of the hotel (hotel property, infrastructure, sanitary safety measures, service quality, etc.) of the quarantined population and their post-stay behavioral intentions (Alhamidi and Alyousef, 2020; Pratt and Tolkach, 2022). However, little research has been conducted on potential consumers without quarantine experiences regarding their attitudes toward QHs and behavioral intentions.

In fact, the COVID-19 outbreak disrupted daily operations and even threatened the survival of the hotel industry. The quarantine hotel’s initiative to take on the quarantine operations and voluntarily provide accommodation for medical staff or those in need of quarantine is a way for the hotel industry to actively give back to the community in this public health crisis and is a win-win CSR marketing strategy (Teng et al., 2021). Although COVID-19 has temporarily promoted the realization of CSR strategies in the hotel industry, this study believes it necessary to delve into how the post-pandemic era can lead to more beneficial CSR marketing strategies for hotel industry stakeholders, especially potential consumers.

### CSR and hotel consumer behavior

CSR refers to the economic, legal, ethical, and philanthropic responsibilities that companies need to take towards the wider society and internal and external stakeholders beyond their own interests, e.g., government agents, suppliers, consumers, staff, communities, and the environment, and corporate actions to further improve social welfare (Carroll, 1999; McWilliams and Siegel, 2001). The hotel industry often faces issues such as long working hours, high staff turnover rate, and serious food waste. Therefore, international hotel brands, including Hilton, Starwood, and Boutique, have incorporated CSR into their business philosophies and regularly issue CSR-related reports to the public to demonstrate their commitment to social issues (Ahn and Shiwen, 2022). In addition, hotels also link their corporate image with their response to social needs and strive to create a trustworthy corporate image for their customers in order to obtain a competitive advantage in the market (Lo, 2020). For example, research has found that CSRs undertaken by hotels can positively affect consumers’ willingness to purchase hotel products in the future (Boronat-Navarro and Pérez-Aranda, 2019). Therefore, CSR strategy plays a crucial role in hotel development.

Major crisis events like COVID-19 are the real CSR test for hotels. Operating pressures during a crisis often drive companies to pursue short-term gains and reduce long-term investments such as CSRs (Rhou and Singal, 2020; Qian et al., 2021). Prior to COVID-19, only a few studies had focused on hotels’ undertaking of CSRs in crises. For example, in response to the 2004 Indian Ocean earthquake and tsunami, local hotels in Phuket actively fulfilled a range of CSRs, including providing accommodation for rescue workers, helping with rescue and rebuilding efforts in the affected areas, and providing relief materials to those affected by the disaster (Henderson, 2007). With the outbreak of COVID-19, increasingly more attention has been attracted to different themes of CSR in the hotel industry during crises, such as the undertaking of CSRs (Hao et al., 2020), the impact of CSRs on corporate business performance (Shin et al., 2021), and the behavioral responses of staff and consumers to CSRs (Goh and Baum, 2021; Tong et al., 2021). Existing research on hotel CSRs during crises is still in its early stages. Previous studies have drawn inconsistent conclusion and the inconsistency needs to be solved (Shin et al., 2021; Tong et al., 2021). Specifically, there was a negative impact of hotel CSR on the booking intention (BI) of potential consumers and market value (Shin et al., 2021). However, social exchange theory suggests that if all parties are treated as equals in a social exchange process between two or more groups or individuals, the outcome will be satisfactory (Olever, 1997). The existing studies have incorporated the social exchange theory with CSR activities and have involved positive consumer response behaviors in social exchanges, such as organizational trust, customer satisfaction, and loyalty (Rather, 2018). According to the social exchange theory, when potential customers perceive that the benefits they can obtain from hotel CSR behaviors far outweigh the costs, they will be satisfied and will be motivated to show strong reciprocal willingness (Palmatier et al., 2009; Bock et al., 2016). Sudies on hotels during the pandemic also showed that CSR contributions of hotels enhanced BI of potential consumers (Tong et al., 2021).

In summary, the previous studies have several limitations: First, most previous studies on hotel social responsibility focused on its importance, effects, and stakeholder perceptions (Qian et al., 2021), while ignoring the differences in the response of potential consumers to CSR behaviors of different hotels. As the most important stakeholders of hotel CSRs, the different behaviors of potential consumers in response to hotel CSR practices before and after COVID-19 are worth further investigation. Second, in previous studies, the behavior of providing quarantine services was regarded as a manifestation of the willingness of hotels to undertake social responsibility, but there is little research on the effect of CSR behaviors of QHs on BI of potential consumers and the involved mediating mechanisms (Shin et al., 2021; Tong et al., 2021). During the COVID-19 pandemic, quarantine hotels providing accommodation for medical staff, suspected cases, travelers under medical observation, and returning guests is a demonstration of their CSRs in the crisis (Teng et al., 2021). These actions may encourage potential consumers to trust the hotel brand, and potential consumers may be grateful for the hotel’s hard work during the crisis, thus increasing consumers’ reservation intention of the hotel’s products (Boronat-Navarro and Pérez-Aranda, 2019). Third, in previous studies, the researchers did not specify the mechanism of behavioral response of potential consumers to the engagement of hotels in CSR activities (e.g., taking the quarantine business) under significant harm scenarios. Existing theoretical studies have not fully explained how hotel CSRs affect consumer reservation behavior during crises (Shin et al., 2021; Tong et al., 2021). Specifically, if a hotel takes on quarantine operations in a crisis, some potential consumers may consider the hotel at risk and, in turn, not book the hotel. It is also possible that some consumers will be more willing to book the hotel because they are grateful for the hard work it has put into the community during the crisis. Given that potential consumers are the most important stakeholders in hotel CSRs, consumers’ reservation intention plays an important predictive role in the occurrence of consumer behavior (Guzzo et al., 2020; Shin et al., 2021). Therefore, this study believes that the behavioral response mechanism of potential consumers to hotels’ involvement in CSR activities during a crisis is a key issue in understanding the impact of hotel CSR on corporate marketing strategies and long-term competitiveness.

## Study design

Based on CSR theory and social exchange theory, we examined the response mechanism of potential consumers to the socially responsible behavior of QHs using mixed methods, focusing on QH BI of potential consumers after COVID-19 ends. In Study 1, we explored the perception of potential consumers on QHs and their intention to book such hotels after the pandemic using the qualitative method based on grounded theory to construct the theoretical model of QH BI of potential consumers and to formulate research propositions. After the literature extrapolation of the conceptual framework derived from Study 1, the empirical hypotheses were proposed and would be verified in Study 2. In Study 2, the influencing mechanisms of QH BI of potential consumers were examined using two situational experiments to identify the specific influencing mechanisms. Quantitative path analysis was performed in Study 2 using PROCESS macro (3.1), a program that can be used to test the regression analysis of indirect (mediated) effects. The program was selected for the analysis due to multiple reasons. Firstly, it is an easy-to-use software that can analyze various research models for SPSS users. Secondly, the regression coefficient results differ very little under the large sample size and observed variable settings compared to the structural equation model (Hayes et al., 2017). Thirdly, when testing for mediating effects, Non-parametric percentile Bootstrap methods that come with the software have higher testing power (Hayes and Scharkow, 2013).

## Study 1: Qualitative study on QH BI of potential consumers from a CSR perspective

Study 1 explored the formation path of QH BI of potential consumers from a CSR perspective. Grounded theory is an effective method for constructive research to specify the formation processes of social cognition and social psychology (Glaser et al., 1968). Therefore, in the first phase of this study, the material was collected through semi-structured interviews and processed with the grounded theory to construct a model of the formation mechanism of QH BI of potential consumers.

### Samples and data collection

In Study 1, the interviewees were between 20 and 40 years old and their occupations are students, civil servants, hotel managers, teachers, etc. They mainly come from Chongqing, Shanghai, and Hangzhou, and all had booked hotels within the last year. The interviews span from December 2021 to March 2022. Due to COVID-19, the interviews were mainly conducted by videoconferences. The questions in the interview outline include personal information of the interviewee (e.g., age, occupation, etc.), his/her understanding of QHs and CSR of QHs, his/her intention to book QHs (vs. NHs) and the factors influencing his/her decision. The concept of QH was explained to the interviewees so as to avoid the possible influence of the inconsistencies in their understanding on the study. The actual research process follows the principle of theoretical saturation (Glaser et al., 1968). Information saturation was reached after interviewing 17 participants, and no new basic concepts were found in the primary coding. The research process stopped until the end of the interview of the 21st participant. To facilitate the analysis, the 21 participants were coded in the form of “T*,” which represents their serial number. The average interview time for each participant was 32 min. In accordance with the consensual qualitative research (Alhamidi and Alyousef, 2020), 840 valid interview sentences were obtained.

### Material analysis

Following the principle of theoretical saturation level, 3/4 of the interview transcripts were randomly selected for coding analysis and model construction, and the remaining 1/4 of the interview transcripts were reserved for theoretical saturation level verification (Rubin and Rubin, 2011). The grounded theory analysis was performed by open coding, axial coding and selective coding of the material obtained from the research (Pandit, 1996).

#### Open coding

Open coding is the process of material collection and analysis, in which the content of the material is indicated by identifying and then naming concepts and categories. At this stage, the collected material was abstracted into concepts for frequency counting, and the concepts with a high frequency of occurrence were retained (those that occurred twice or more were retained, and those that occurred only once but were very important were retained). By continuous decomposition, comparison, conceptualization and the mining of categories, we finally obtained 106 concepts and 12 categories.

#### Axial coding

Axial coding refers to the division of categories based on in-depth analysis and repeated comparison of their causal relationships to form more systematic and general categories. After axial coding, we found interrelationships and logical order among 12 subcategories of the factors influencing QH BI of potential consumers. Following further derivation and generalization, we obtained four main categories, namely, “customer trust (CT),” “customer gratitude (CG),” “CSR perception,” and “individual factor,” which are shown in Table 1.

**Table 1:** Results of axial coding.

Main category	Subcategory	Original statement
CG (14)	Giving back (4)	I am willing to do something to give back to QHs. (T05)
	Gratitude for help (6)	I feel more or less grateful for QHs. (T16)
	Gratitude from the heart (4)	I would feel grateful. (T07)
Individual factor (11)	Risk aversion (8)	I am a risk-averter and will try to avoid risk factors threatening my safety. (T04)
	Companion need (3)	I have a child, and I will prioritize his/her need when reserving hotels and the opinions of my family are also considered; hotels with nature views are ideal. (T20)
	Standard specification (7)	I think the standards for hotels designated as quarantine sites by the government are higher. (T07)
CT (27)	Sanitary guarantee (6)	I would trust QH more in terms of sanitary safety. (T10)
	Reliable quality (5)	I would trust QHs more in terms of hygienic quality. (T11)
	High credibility (9)	The service at QHs is of high quality and reliable. (T13)
	Corporate Image (4)	I admire QHs that bear social responsibility, and such a corporate image attracts me. (T21)
CSR perception (34)	Social responsibility (21)	I feel that it is not easy for hotels to take the quarantine business, which manifests their willingness to take social responsibility (T06)
	Social contribution (9)	QHs show sentimentalism and can contribute to society (T17)

#### Selective coding

**Figure 1:**
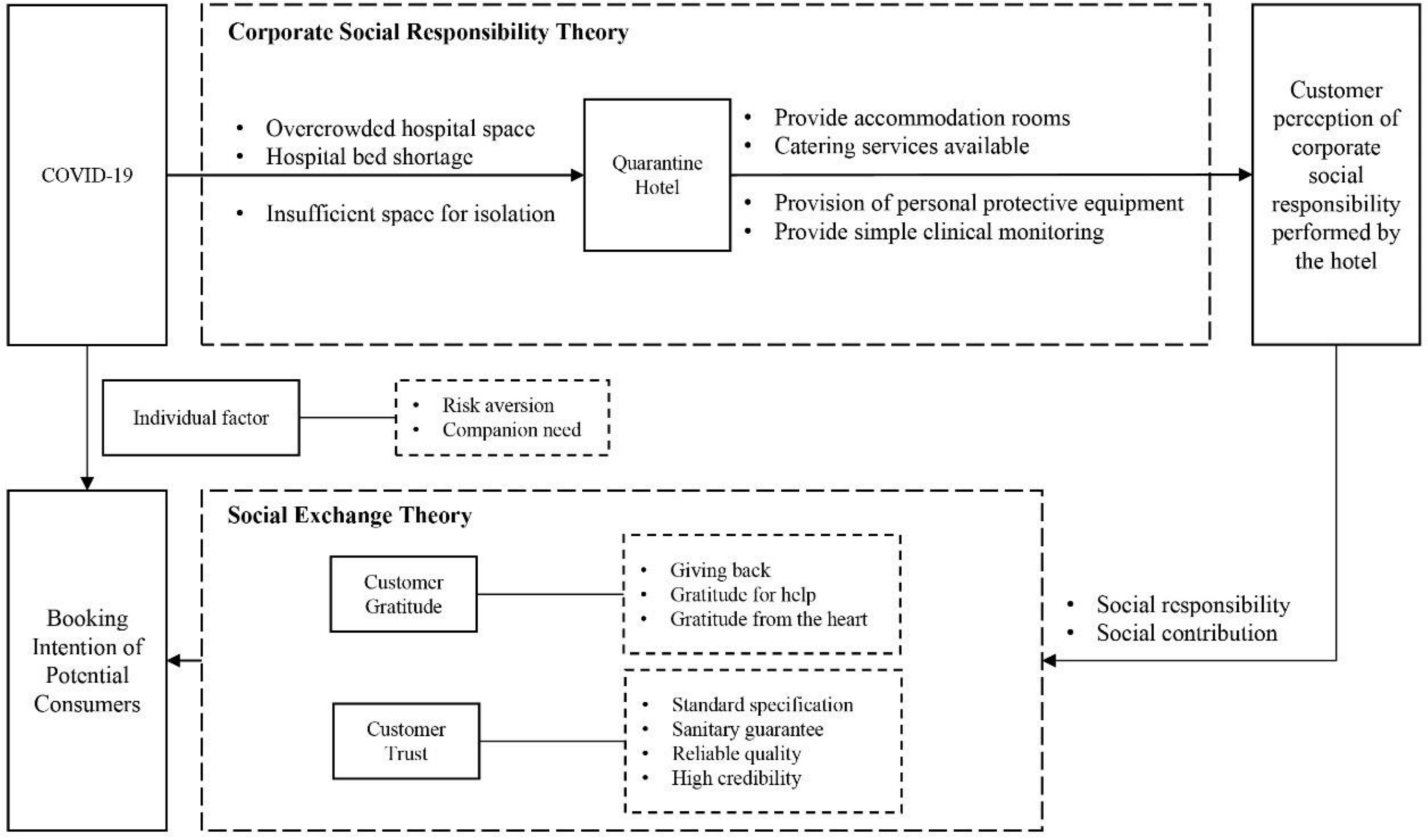
The formation path of potential consumers booking intention of Quarantine Hotel.

Selective coding is axial coding at a higher level of abstraction, and its purpose is to form a complete “story line” by merger and integration based on the core categories. In this paper, based on the theories of hotel CSR, we took “formation QH BI” as the core category to complete the final generalization and integration and related the core category with other main categories to further present the relationship between main categories and the corresponding supporting concepts. We constructed a model of the formation mechanism of QH BI in the form of story line, as shown in Figure 1.

### Model elaboration and logical relationship

The model in Figure 1 shows how various influencing factors affect QH BI of potential consumers. Under the COVID-19 shock, the behavior of providing quarantine services is seen as a hotel CSR behavior and is an important antecedent variable influencing BI of potential consumers. CT and CG, as positive moral-emotional reactions of potential consumers to CSR behavior, explain the intrinsic mechanism of the impact of undertaking social responsibility on QH BI of potential consumers. Customers can perceive CSR of QHs, and they will feel trust and gratitude since the social benefits they get outweigh the cost. These positive emotional responses enhance their QH BI. In addition, we found that hotel property (e.g., location (20), service quality (14), product price (12), hotel brand (9), etc.) and customer attribute (risk aversion (10), companion need (3), etc.) are important factors that influence BI of potential consumers. This finding is similar to that in existing studies (Shin et al., 2021; Sun et al., 2022). In Study 1, the following four propositions were put forward and will be verified in Study 2.

Proposition 1: Potential consumers are more willing to book QHs than NHs.

Proposition 1: The preference of potential consumers for QHs is attributed to CSR.

Proposition 3: CT and CG will affect QH BI of potential consumers and will mediate the influence of CSR on BI of potential consumers.

Proposition 4: QH BI of potential consumers is influenced by individual factors.

## Study 2: Experimental study on the intention of potential consumers to book QHs

Study 2 aims to examine the formation mechanism of QH BI using a quantitative approach. Study 1 showed that CSR behaviors of QHs contribute to QH BI of potential consumers. This finding is not consistent with the conclusion of the existing study (Shin et al., 2021). There is also a study showing that the prepay willingness of consumers varies with the time point of the CSR contribution by hotels (Tong et al., 2021). In this paper, we understood the formation mechanism of QH BI of potential consumers within the theoretical framework of CSR theory and social exchange theory; we argued that when potential consumers perceive that the socially responsible behaviors of quarantined hotels during the pandemic bring benefits to the society, they develop positive emotions such as gratitude and trust and give back to the hotels by purchasing or other behaviors (Gouldner, 1960; Freeman, 2010). This paper focuses on the difference in QH BI and NH BI of potential consumers when the COVID-19 pandemic recedes. Since this scenario did not occur, the experimental study was performed under the simulated scenario.

### Hypothesis development

#### QH, CSR perception, and BI

Faced with COVID-19, the hotel industry actively responded to the government’s call and consciously took quarantine tasks to fight against the pandemic. These actions are a manifestation of the hotels’ active commitment to CSR (Gürlek and Kılıç, 2021).Actively undertaking social responsibility can contribute to good brand perception and a positive corporate image, which will influence the behavioral intentions of customers (Perez and Rodriguez del Bosque, 2015). Based on the above analysis, the following hypothesis is proposed:

*H1*: Potential consumers are more willing to book QHs (vs. NHs).

In the face of the fear and uncertainty brought by COVID-19, consumers have become more concerned about the pandemic prevention and control measures hotels take to reduce health risks (Sun et al., 2022). CSR initiatives can help hotels gain positive evaluation and recognition by consumers such as loyalty, public praise, and brand attachment (Brown and Dacin, 1997; Sen and Bhattacharya, 2001; Lichtenstein et al., 2004; Marin et al., 2009)Actively fulfilling social responsibility and improving sanitary conditions will contribute to CT, customer satisfaction and BI (Rhou and Singal, 2020; Handani et al., 2022). When potential consumers perceive that QHs are actively fulfilling their social responsibility and believe that they can benefit from such CSR behaviors, they tend to act in a way that benefits the hotel, i.e., hotel BI. Based on the above analysis, the following hypothesis is proposed:

*H2*: The CSR perception of potential consumers mediates the influence of the operation as a quarantine facility on their QH BI. QHs increase the perception of potential consumers on their CSR by providing quarantine services, enhancing BI of consumers.

#### Dual mediating effect of CSR perception, CG and CT

CG behaviors have an incentive value and can promote social relationships by supporting reciprocal or pro-social behaviors between givers and recipients (McCullough et al., 2001; Bartlett and DeSteno, 2006). CSR behaviors have a positive effect on CG, corporate image and consumer behavioral intention (Fernández-Ferrín et al., 2021). CG has a positive influence on behavioral loyalty and immediate purchase intentions (Dewani et al., 2016)Under the attack of COVID-19, QHs undertook pandemic prevention and control tasks and took measures, which reflects their willingness to assume social responsibility. When customers feel grateful for the socially responsible behavior of QHs during the pandemic, they will express their gratitude by reinforcing their behavioral intentions and motivate themselves to give back to QHs. Based on the above analysis, the following hypothesis is proposed:

*H3a*: CG will positively mediate the influence of CSR perception on BI of potential consumers.

Trust is “the expectation of ethical behavior” (Hosmer, 1995)CSR is a form of ethical capital with trust as its core (Godfrey, 2005). CT has a mediating role in the framework of CSR assessment (Vlachos et al., 2009). Some studies have found the mediating role of CG, CT and customer satisfaction in the relationship between CSR and consumer loyalty (Park et al., 2015; Ahn and Kwon, 2020). From a moral perspective, consumers will regard the participation of QHs in CSR activities as a well-intentioned behavior, and this behavior helps establish CT, which is an important antecedent variable affecting consumers’ willingness to purchase. Therefore, we can predict CT toward QHs significantly affects BI. Based on the above analysis, the following hypothesis is proposed:

*H3b*: CT positively mediates the effect of CSR perception on BI of potential consumers.

**Figure 2:**
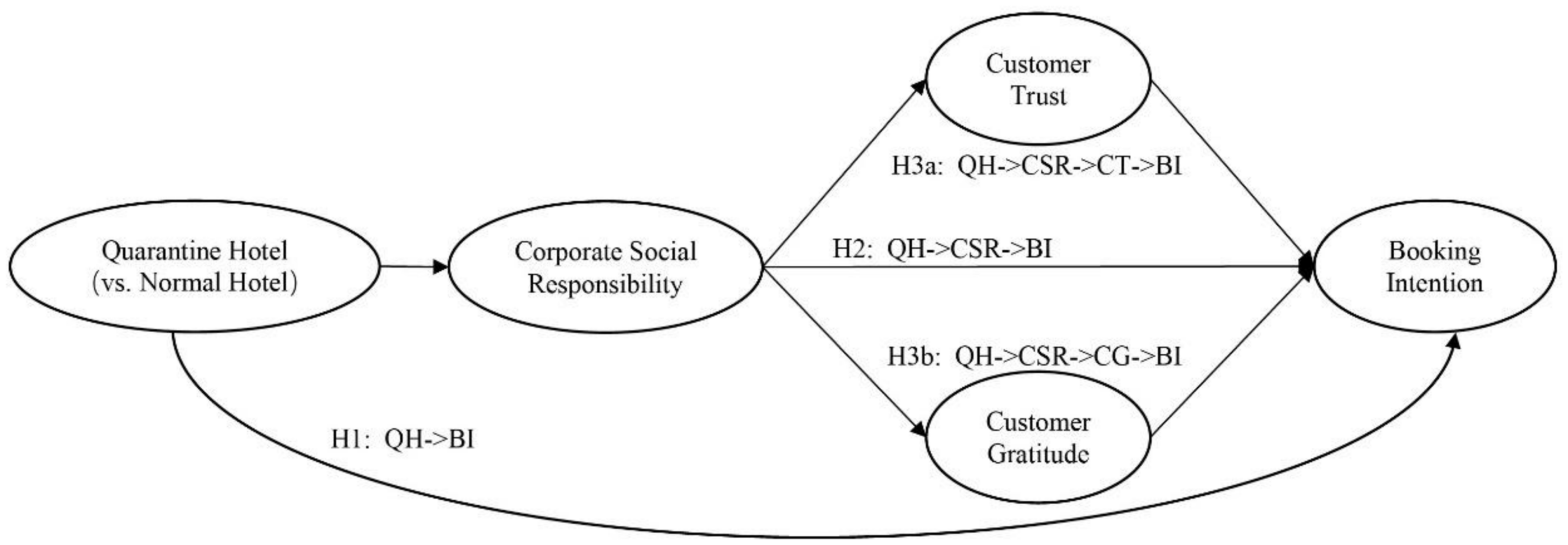
Proposed model.

In this paper, the above research hypotheses were tested by two situational experiments. The specific model is shown in Figure 2. In Experiment 1, we tested the effect of the operation as QHs on customers’ purchase intention, i.e., H1 and H2; In Experiment 2, we tested the mediating role of CSR perception, CT, and CG in this effect, i.e., H3.

### Study 2A

#### Method

Experiment 1 is a one-factor two-level (QH group vs. NH group) between-group experiment, and this experiment aims to test QH BI of potential consumers, in other words, to verify H1 and H2. Prior to the formal experiment, the manipulation check for the validity of the differences in the perception of the experimental material was performed.

#### Participants and stimuli

In this study, the experiment was initiated with a textual scenario. The participants were asked to read the provided information and immerse themselves in the experimental scenario to complete the test. The scenario was described as “You (the participant) plan to book a hotel for your trip after the COVID-19 pandemic is over.” The description of QH and NH is shown in Appendix 1. Each participant was provided with one set of experimental materials randomly. The participants answered the questions in the questionnaire after browsing the provided material.

The questionnaire consisted of two parts. The first part was composed of manipulation control questions; the participants were asked to answer “to what degree is this hotel a quarantine one” (7-level scale, 1 = regular hotel, 7 = QH), and the second part includes questions related to demographic characteristics.

We recruited 80 participants with hotel accommodation experience at a university for a fee (¥1) and obtained 74 valid samples (47.3% male, *M*_age_ = 20.421, *SD = 1*.108). The results showed that the inter-group difference in the mean score of hotel type evaluation was significant (*M*_quarantine_ = 5.526, *SD*_quarantine_ = 1.333; *M*_control_ = 4.583, *SD*_control_ = 1.180), and the one-way ANOVA results showed that the difference between the two groups was significant [*F*(1,72) = 12.300, *p <* 0.001], indicating that the manipulation of the differences in the perception of the experimental materials was successful and the materials could be used in the formal experiment.

#### Data collection and procedures

The materials for the formal experiment were the same as those for the pre-test. For Experiment 1, we recruited 100 enrolled students with hotel accommodation experience at a university for a fee (¥2). These participants were randomly divided into two experimental groups (QH group and NH group). The researchers informed the participants that the purpose of the experiment was to investigate their QH BI after the end of the COVID-19 pandemic. The participants were asked to read the provided situational material at their habitual speed and then fill out a questionnaire about their perception of hotel social responsibility and BI (shown in Appendix 2; Dodds et al., 1991; Wagner et al., 2009). Subsequently, the participants wrote down the answers to questions related to demographic characteristics. A total of 100 questionnaires were collected in Study 2A, and 98 valid questionnaires were obtained by excluding 2 invalid questionnaires.

#### Results

The results of the independent samples *t-*test of the manipulation check showed a significant inter-group difference in the hotel type evaluation score (*M*_quarantine_ = 5.521, *SD*_quarantine_ = 1.237; *M*_control_ = 4.080, *SD*_control_ = 1.397), and the difference in the mean evaluation scores of the QH group and the NH group was 1.441, *t = 5*.395; *p <* 0.001, indicating that the manipulation of hotel type was successful.

The results of one-way ANOVA showed a significant inter-group difference in BI (*α =* 0.947), (*M*_quarantine_ = 5.526, *SD*_quarantine_ = 0.940; *M*_control_ = 4.425, *SD*_control_ = 1.214; *F*(1,96) = 25.070, *p* < 0.001), indicating that potential consumers were more willing to book QHs, effectively verifying H1. The inter-group difference in hotel social responsibility perception of customers (*α* = 0.890) was also significant, (*M*_quarantine_ = 5.526, *SD*_quarantine_ = 0.940; *M*_control_ = 4.425, *SD*_control_ = 1.214; *F*(1,96) = 25.070, *p* < 0.001).

Mediating effect test: The independent variable was set to be “0” for “NH” and “1” for “QH” (the same in Study 2B below). The Bootstrap method was used to test the mediating effect of customer perception of hotel social responsibility (PROCESS, Model 4, sample size 5000, confidence interval 95%; Hayes et al., 2017). The results proved that the mediating effect of customer perception of hotel social responsibility was significant (*β = 0*.60, LLCI *=* 0.230, ULCI *=* 1.039, excluding 0), verifying H2.

### Study 2B

#### Method

To further investigate whether there are multiple mediating mechanisms in the relationship between hotel CSR and consumer BI, we conducted Study 2B, a one-factor two-level (QH group vs. NH group) between-group experiment, in which multiple mediating variables (CT and CG) were introduced based on Study 2A.

#### Participants

For Experiment 2, we recruited 290 students at eight universities in five provinces of China, including Fujian, Yunnan, Shandong, Hainan, and Guangdong, for a fee (¥3). Seven sets of invalid data were excluded, and 283 valid questionnaires were obtained (48.8% male, *M*_age_ = 21.38, *SD* = 2.173).

#### Data collection, design, and procedures

The participants were randomly divided into the QH group and NH group and provided with corresponding experimental materials. The experimental scenario and information description were the same as those in Study 2A. The participants answered the questions in the questionnaire after browsing the provided material. They were asked, “To what degree is the hotel a quarantine one” (7-level scale, 1 = NH, 7 = QH). Then their intention to book the tested hotel and their perception of hotel social responsibility were measured (question items were the same as those in Experiment 1). Subsequently, their trust (Gibreel et al., 2018) and gratitude (Mangus et al., 2017) toward the hotel were measured, respectively, as specified in Appendix 2. Finally, the participants wrote down the answers to the questions related to demographic characteristics.

#### Results

The results of the independent samples *t-*test of the manipulation check showed a significant inter-group difference in the hotel type evaluation score (*M*_quarantine_ = 5.036, *SD*_quarantine_ = 1.738; *M*_control_ = 2.958, *SD*_control_ = 1.705), and the difference in the mean evaluation scores of the QH group and the NH group was 2.077, *t =* 10.147; *p <* 0.001, indicating that the manipulation of hotel type was successful.

The results of the one-way ANOVA showed the difference in BI (*α =* 0.948) between groups was significant (*M*_quarantine_ = 5.015, *SD*_quarantine_ = 1.155; *M*_control_ = 4.650, *SD*_control_ = 1.168; *F*(1,281) = 6.958, *p* = 0.009), and the inter-group difference in customer perception of hotel social responsibility (*α =* 0.909) was also significant (*M*_quarantine_ = 5.474, *SD*_quarantine_ = 1.027; *M*_control_ = 4.816, *SD*_control_ = 1.167; *F*(1,281) = 25.071, *p* < 0.001). These results were consistent with those of Study 2A.

Mediating effect test: The independent variable was set to be “0” for “NH” and “1” for “QH.” The Bootstrap method was used to test the multiple mediating effects of customer perception of hotel social responsibility and CT/CG (PROCESS, Model 81, sample size 5000, confidence interval 95%; Hayes et al., 2017), as shown in Table 2. We found that the direct effect of hotel type on BI was significant (LLCI *=* 0.137, ULCI *=* 0.317, excluding 0), and the mediating effect was significant (LLCI *=* 0.103, ULCI *=* 0.489, excluding 0), indicating that the mediating variables play a partially mediating role. Specifically, the mediating path of “QH→CSR→BI” is significant (LLCI *=* 0.173, ULCI *=* 0.349, excluding 0). These results were consistent with those in Study 2A. The mediating paths “QH→CT→BI” (LLCI = −0.105, ULCI = 0.009, including 0) and “QHT→CGT→BI” (LLCI = −0.062, ULCI = 0.145, including 0) were not significant. The mediating paths “QHT→CTT→BI” (LLCI = 0.033, ULCI = 0.213, excluding 0) and “QHT→CGT→BI” (LLCI = 0.10462, ULCI = 0.370, excluding 0) were significant. H3 was verified.

**Table 2:** The pathway mediating the influence on QH BI of potential consumers.

Type of effect	Specific pathway	Effect	*se*	*LLCI*	*ULCI*
Direct effect	QH→BI	0.090	0.115	0.137	0.317
Mediating effect	QH→CSR→BI	0.056	0.057	0.173	0.051
	QH→CT→BI	0.042	0.029	−0.105	0.009
	QH→CG→BI	0.039	0.052	−0.062	0.145
	QH→CSR→CT→BI	0.121	0.046	0.033	0.213
	QH→CSR→CG→BI	0.222	0.069	0.104	0.370

In summary, Experiment 2 showed that QH BI of potential consumers would change with their perception of hotel social responsibility. The reason is that the social responsibility perception of consumers affects their trust and gratitude toward hotels, which further influences their BI.

## Discussion and conclusions

### General discussion

Customer acceptance is an important factor influencing the decision of hotel managers whether to participate in pandemic prevention and control activities. Therefore, it is extremely important to identify factors influencing customer acceptance of QHs. The present research was conducted with mixed methods. The extent to which potential consumers are willing to book QHs was investigated and novel mediating mechanisms for QH BI were found.

In qualitative Study 1, we found that potential consumers believed that hotel booking during COVID-19 would be influenced by hotel factors and individual factors. Potential consumers would favor QHs since such hotels fulfilled more CSR, which is consistent with the finding in previous studies (Handani et al., 2022; Sun et al., 2022), further confirming the idea that hotels fulfilling their social responsibility during the pandemic will be recognized by consumers. Based on the findings of the qualitative study, we conducted Study 2 to examine the differences in QH BI and NH BI of potential consumers as well as the internal mechanisms. In Study 2A, we found that potential consumers would be more willing to book QHs than NHs and more likely to perceive CSR of QHs. In Study 2B, we further found that potential consumers perceived that QHs fulfilled more CSR than NHs, making potential consumers show more trust and gratitude towards QHs.

### Theoretical implications

The findings in this paper complement the research on the impact of providing quarantine services on the behavior of potential consumers and the research on hotel social responsibility.

First, our study explores the impact of QH CSR on BI of potential consumers, extending the application scenario of CSR. Most previous studies focused on the relationship between hotel social responsibility and internal stakeholders (e.g., customers and employees; Rhou and Singal, 2020; Qian et al., 2021) and confirmed the positive effect of hotel social responsibility on consumer behavioral intention (Lo, 2020). These studies were conducted mainly under normal scenarios, while studies on the effects of socially responsible behaviors of hotels under major crisis events like COVID-19 were rare (Hao et al., 2020; Rhou and Singal, 2020). This paper focuses on the effect of hotel participation in CSR activities (e.g., undertaking quarantine tasks) on BI of potential consumers under the scenario of the COVID-19 pandemic. The results confirm that the operation as QHs (vs. NHs) reinforces BI of consumers by arousing their sense of trust and gratitude. In addition, we examined the underlying mechanism of the influence of the socially responsible behavior of QHs on BI of potential consumers with mixed methods. This research concludes that hotel CSR behavior under major crisis events positively influences BI of potential consumers and enriches relevant research on hotel social responsibility, which also provides an empirical basis for exploring the effects of hotel CSR practices under major crises (Qian et al., 2021; Tong et al., 2021).

Second, our research addresses the contradiction between the conclusions in previous studies on the response of potential consumers to socially responsible behaviors of QHs. In this research, we argued from the perspective of social exchange theory that in the case of a major event such as COVID-19, the socially responsible behaviors of hotels bring significant benefits to potential consumers, and potential consumers form a high-quality social exchange relationship with QHs, which leads to a stronger reciprocal willingness of potential consumers when the pandemic recedes. This conclusion is different from that in the previous study that CSR behaviors have a negative effect on BI of potential consumers (Shin et al., 2021). This research supports the idea that hotel CSR behavior positively influences BI of potential consumers (Tong et al., 2021). Combining CSR theory with social exchange theory enriches the theoretical explanation of hotel consumer behavior in major crises (Marin et al., 2009; Lo, 2020), which provides a theoretical basis for the development of marketing strategies for QHs in the post-pandemic era(Teng et al., 2021).

Third, our research clarifies the two mediating mechanisms involved in the effect of CSR of QHs on potential consumers’ BI. Previous literature explored the mechanisms of the response of potential consumers to socially responsible behavior of hotels (Gao and Mattila, 2016; Ahn and Kwon, 2020), but the specific paths of response mechanisms have not been clarified. In the present research, where the qualitative approach and quantitative approach were combined, we found the dual mediating mechanisms for the effect of the operation as QHs on BI of potential consumers. The mechanism is different from the previous impact mechanism of hotel CSR practices in common scenarios (Lichtenstein et al., 2004; Marin et al., 2009). Potential consumers are more likely to perceive CSR of QHs (vs. NHs) and internalize the socially responsible behavior as their perceived moral benefit, which will enhance their moral identity as well as trust and gratitude towards QHs, and then contribute to strong QH BI (Romani et al., 2013). This finding complements the research on the response mechanisms of potential consumers to socially responsible behaviors of hotels (Rhou and Singal, 2020; Qian et al., 2021) and provides a theoretical direction for future research on marketing strategies of QHs from an ethical perspective.

### Managerial implications

During the COVID-19 pandemic, QHs play an important role, and their survival and development in the post-epidemic era and the further future deserve attention. This paper provides several recommendations for the development of QHs. First, hotels do not need to worry about losing market share after providing quarantine services as the present research shows that potential consumers are more willing to book QHs considering that the QHs take more CSR and sanitary standards in QHs are higher than in NHs. This finding provides guidance for the participation of hotels in COVID-19 prevention and control activities. Second, BI of potential consumers is determined by their perception of the social responsibility of QHs. Potential consumers will trust the service quality of QHs and be grateful for their contribution. It is indicated that CT and CG both arise from CSR behaviors. QHs should pay attention to the propaganda of the fulfillment of their social responsibility so as to win the trust of potential consumers or members. Of course, while actively booking quarantine hotels to complement their undertaking of CSRs, potential consumers also practice rational consumption to prevent service quality decline, poor hygiene conditions, and excessively high product prices. Third, CT and CG positively impact QH BI of potential consumers. Therefore, managers of QHs should focus on the propaganda of CSR behaviors from an ethical perspective to facilitate emotional resonance in potential consumers and lay the emotional basis for the recovery and marketing promotion of the hotels.

### Limitations and future research directions

This research has some limitations and the future research directions are as follows. First, this research focuses on QH BI of potential consumers. We suggest that in the future, researchers could explore the revisiting willingness of consumers with QH accommodation experience and compare the findings with the conclusion of the qualitative study in the present research to extend the practical implications of the involvement of the hospitality industry in pandemic prevention and control activities. Second, although we achieved strong internal validity by using mixed methods and increased the external validity of the findings with experimental studies, the present research still has certain limitations, such as the difficulty in the full control of other variables (e.g., risk perception). In the future, field studies can be performed to test the reproducibility of the findings for greater external validity. Third, in this research, we used self-reported cross-sectional data for the quantitative study, which may lead to general response bias since some respondents tend to inaccurately answer the survey questions. In the future, researchers could use longitudinal real hotel booking consumption data on the OTA platform to examine the differences between QH and NH booking behaviors. Fourth, in addition to performing a longitudinal study, researchers could analyze the potential moderating/mediating role of some variables, such as cultural differences. Finally, this study was conducted in a Chinese cultural context. The collectivist characteristics of Chinese culture may constitute cultural differences from the cultural contexts in the existing literature. Future research could focus on comparing the behavioral differences of potential consumers in different cultural contexts in response to hotel CSRs during major crises.

## Data availability statement

The original contributions presented in the study are included in the article/Supplementary material, further inquiries can be directed to the corresponding author.

## Author contributions

GW contributed to conception and design of the study, performed the statistical analysis and wrote the first draft of the manuscript. YW and XL wrote sections of the manuscript. ML contributed to conception and design of the study. All authors contributed to the article and approved the submitted version.

## Conflict of interest

The authors declare that the research was conducted in the absence of any commercial or financial relationships that could be construed as a potential conflict of interest.

## Publisher’s note

All claims expressed in this article are solely those of the authors and do not necessarily represent those of their affiliated organizations, or those of the publisher, the editors and the reviewers. Any product that may be evaluated in this article, or claim that may be made by its manufacturer, is not guaranteed or endorsed by the publisher.
