# Supplementary material for: Formation mechanism of the quarantine hotel booking intention of potential consumers
Source: Front Psychol. 2022 Sep 28;13:997667. doi: 10.3389/fpsyg.2022.997667 (PMC9555081; doi:10.3389/fpsyg.2022.997667)
Supplement: Supplementary file 1 [file Data_Sheet_1.docx]

***Appendix 1：The description of QH and NH***

| Normal Hotel | Xiangyue Hotel is a comprehensive hotel integrating catering, accommodation, entertainment, tourism and business.  With an elegant and comfortable environment, the hotel has well-equipped facilities and simple but stylish rooms which provides every guest with a sound sleep. The hotel also has convenient transportation, with the airport shuttles, a subway station and a bus station just downstairs.  Facing the severe situation of epidemic prevention, we have always maintained a professional and high-quality service level, actively cooperated with epidemic prevention policies, and guided employees and customers to increase their awareness of epidemic prevention. Meanwhile, we have been adhering to the mission of "Saving lives is of paramount importance" and "Go where there is epidemic; fight it till it perishes", and sticking to the "Customer first; service first" mindset, which helps to win us wide recognition from the society. |
| --- | --- |
| Quarantine  Hotel | Xiangyue Hotel is a comprehensive hotel integrating catering, accommodation, entertainment, tourism and business.  With an elegant and comfortable environment, the hotel has well-equipped facilities and simple but stylish rooms. During the epidemic situation, the hotel actively participated in the anti-epidemic campaign. In addition to carrying out all -round disinfection of various regions, the hotel promptly carried out room reconstruction projects to observe the requirements of a quarantine hotel and began to receive inbound travelers to undergo compulsory quarantine.  During the continuous participation in the anti-epidemic campaign, we have received 2,789 guests from home and abroad, 169 medical and healthcare workers, 105 police officers, 148 anti-epidemic hotel personnels, and they have performed 0 suspected case, 0 infection, and 0 confirmed case. By practicing the service tenet of "warm hospitality stems from our nature"，we have achieved good social benefits. |

***Appendix 2：The Questionnaire of Study2A and Study2B***

| ***CSR-Corporate Social Responsibility (3 items)*** | very high 🡪 very low | | | | | | |
| --- | --- | --- | --- | --- | --- | --- | --- |
| In my opinion, this hotel is a socially responsible hotel. | 7 | 6 | 5 | 4 | 3 | 2 | 1 |
| In my opinion, this hotel is concerned to improve the well-being of society. | 7 | 6 | 5 | 4 | 3 | 2 | 1 |
| In my opinion, this hotel follows high ethical standards. | 7 | 6 | 5 | 4 | 3 | 2 | 1 |
| ***CT-Customer Trust***  ***(4 items)*** | very high 🡪 very low | | | | | | |
| I think this hotel is trustworthy | 7 | 6 | 5 | 4 | 3 | 2 | 1 |
| I think this hotel can be trusted | 7 | 6 | 5 | 4 | 3 | 2 | 1 |
| I think this hotel is honest and reliable | 7 | 6 | 5 | 4 | 3 | 2 | 1 |
| I think this hotel is keeping its promises | 7 | 6 | 5 | 4 | 3 | 2 | 1 |
| ***CG-Customer Gratitude***  ***(3 items)*** | very high 🡪 very low | | | | | | |
| I feel grateful to this hotel | 7 | 6 | 5 | 4 | 3 | 2 | 1 |
| I feel thankful to this hotel | 7 | 6 | 5 | 4 | 3 | 2 | 1 |
| I feel appreciative of this hotel | 7 | 6 | 5 | 4 | 3 | 2 | 1 |
| ***BI-Booking Intention***  ***(4 items)*** | very high 🡪 very low | | | | | | |
| The likelihood of purchasing this hotel | 7 | 6 | 5 | 4 | 3 | 2 | 1 |
| If I were going to book a hotel, I would consider booking this hotel | 7 | 6 | 5 | 4 | 3 | 2 | 1 |
| The probability that I would consider booking the hotel | 7 | 6 | 5 | 4 | 3 | 2 | 1 |
| My willingness to book the hotel | 7 | 6 | 5 | 4 | 3 | 2 | 1 |
